# Heavy Metal Contamination: Sources, Health Impacts, and Sustainable Mitigation Strategies with Insights from Nigerian Case Studies

**DOI:** 10.12688/f1000research.160148.1

**Published:** 2025-01-27

**Authors:** Babafemi Laoye, Peter Olagbemide, Tolulope Ogunnusi, Oghenerobor Akpor

**Affiliations:** 1 Department of Biological Sciences, Afe Babalola University, Afe Babalola University, Ado-Ekiti, Ekiti, 360101, Nigeria

**Keywords:** Heavy metal; Phytoremediation; DNA; Cadmium; Lead; Arsenic; Mecury Anthropogenic; Bioaccumulation; Bio-magnification.

## Abstract

Heavy metal contamination has gradually become a very much important significant global issue due to its continual existence in the environment and bioaccumulation in the ecosystems, posing deleterious risks to human health. This review aims to investigate the sources, pathways, and toxicological impacts of heavy metals such as cadmium, lead, mercury, and arsenic, elucidating their health consequences and plausible mitigation strategies. Furthermore, the review explores the dual origins of heavy metal contamination; natural geological processes and anthropogenic activities such as industrial emissions, mining, and agricultural practices. These heavy metals sip into soil, water, and food chains, leading to bioaccumulation, bio-magnification and causing significant health risks, including cardiovascular diseases, neurological disorders, and reproductive toxicity. Additionally, the addition of indigenous case studies from Nigeria, such as lead poisoning in Zamfara State and contamination in the Great Kwa River of Cross Rivers State underscores the disproportionate impact of heavy metal pollution in developing nations. These case studies reveal the socio-economic and environmental dimensions of the issue, providing a contextual understanding of region-specific vulnerabilities and health outcomes. To address these problems, the review evaluates already existing mitigation strategies, including chelation therapy and phytoremediation, while proposing sustainable, cost-effective solutions for reducing exposure and mitigating impacts. It emphasizes the importance of integrative approaches involving policy, community engagement, and technological innovations to fight heavy metal contamination effectively. In conclusion, this seminar contributes to the understanding of heavy metal toxicity, giving and showcasing very much important insights into the sources and health implications of contamination. By integrating theoretical perspectives with practical solutions, this review provides a robust framework for informing policy makers and advancing sustainable environmental management practices.

## 1. Introduction

Heavy metals are naturally occurring elements with a relatively high density present in the ecosystem ( De Carvalho Machado and Dinis-Oliveira, 2023; Ali and Khan, 2018). They are popularly known as metals of environmental concern ( Kothapalli, 2021; Rahman and Singh, 2020). Heavy metal pollution or contamination in water is one of the most consequential environmental issues ( Mishra *et al.*, 2019). This is due to the sturdiness of heavy metals, which cannot be consumed by the body, and the danger they pose to the health of humans contaminated water with heavy metals is used for irrigating crops, and during the rainy season, the water fills groundwater sources, contaminating the drinking water. Thus, heavy metals present in the crops, groundwater, and drinking water lead to several health risks ( Vielee and Wise Jr, 2023; Vetrimurugan *et al.*, 2017). Heavy metals are stable and do not break down into less toxic products. Heavy metals bio accumulate in the food chain and can therefore have debilitating impacts on the health of humans ( Acharya, 2024). The complex metabolic machinery in our body is affected by heavy metal pollution or toxicity, leading to negative health manifestations such as hemolytic anemia ( Teschke, 2024), nerve damage ( Aljelehawy, 2022), nausea ( Engwa *et al.*, 2019) diarrhea ( Su *et al.*, 2023) and feto-maternal bleeding ( Tabassum *et al.*, 2023). Lead is toxic to the kidney ( Khalaf *et al.*, 2024), liver ( Chen *et al.*, 2023) reproductive and nervous systems of people of all ages. Mainly, lead poisoning in children can cause behavioral and cognitive problems ( Gudadhe *et al.*, 2024). Inorganic arsenic (iAs) is the most prevalent toxic form of arsenic in drinking water, which increases the rate of skin diseases in humans ( Abtahi *et al.*, 2023). Cadmium has been implicated to cause hypercalciuria in humans ( Obaid *et al.*, 2023). A recent review revealed that of this exposure to cadmium in human leads to a 31% increase in risk of lung cancer ( Farahmandian *et al.*, 2024). Moreso, mercury have been implicated to cause kidney diseases in humans as a result of bioaccumulation ( Kumar *et al.*, 2023). Also, occupational exposure to chronic viral infections can lead to skin and nasal ulcers, as well as lung cancer ( Yasmeen and Hafeez, 2023) Heavy metals have also been implicated to cause skin pigmentation ( de Carvalho, 2023) skin tumors ( Fu and Xi, 2020) and dermatitis ( Alam *et al.*, 2019).

Moreso, it is imperative to be abreast of the rationale and significance of this review shedding light on the impacts of “Heavy Metal Contamination on Human Health”. Water is very much the most vigorous arsenal for life, however when water is polluted or contaminated with heavy metals like Mercury (Hg), Chromium (Cr), Cadmium (Cd), Pb (lead), As (Arsenic), and Nickel (Ni) it leads to vulnerable diseases such as neural disorders ( Singh and Sharma, 2024), psychosomatic ( Teschke, 2024; Hosen, 2021) mental retardation in neonates ( Dórea, 2019) tumorigenesis and even death ( Kothapalli, 2021). Heavy metal contamination cannot be overemphasized heavy contamination in the food chain, e.g. in fish, milk, drinking water, and also crops irrigated with polluted water that accumulate heavy metals ( Ugoeze *et al.*, 2021). Research have shown that the irrigation water had not only been responsible for the contamination of the crops but also the water sources in the surroundings of the dumping site areas ( Nyiramigisha, 2021). For example, the potential health hazard due to the consumption of contaminated vegetables may arise when the heavy metals are ingested by the consumer ( Rai *et al.*, 2019), or the soils are contaminated to such an extent that they enter into the food chain, and dietary intake as well as drinking water become further contaminated ( Nkwunonwo *et al.*, 2020).

Heavy metals have received considerable attention as a result of too much utilization in different industries, as well as their debilitating injurious impact on human health and the environment ( Vielee and Wise Jr, 2023; Kaur and Sharma, 2021). Heavy metal contamination in the ecosystem as an effect human health via different routes, including soil, water, and industrial emissions ( Briffa *et al.*, 2020). There is a great need to pin point risk populations, and identify plausible avenues and methodology to ameliorate the debilitating effects of heavy metals in our environment.

## 2. Sources of heavy metal contamination

In the natural ecosystem, heavy metals are introduced to the environment perpetually from bed rocks and volcanic substances as a result of disintegration of rocks (weathering) ( Deng *et al.*, 2022). Contamination of heavy metals in the environment is largely due to human interferences (anthropogenic activities) ( Christophoridis *et al.*, 2019). Anthropogenic heavy metals are derived from (a) industrial activities, including the mining, smelting and refining of ores, steel production, and other metallurgical operations; electrical products manufacturing; product finishing and surface coating such as painting and electroplating; the application of heavy metal-based pesticides; preservation of woods and leather ( Marriage and Gjerde, 2024; Obasi *et al.*, 2022), (b) uncontrolled disposal of effluents and smudges from heavy industries ( Elbasiouny *et al.*, 2021); household disposal of domestic hazardous wastes ( Elbeshbishy and Okoye, 2019) and (c) other sources such as the disposal of domestic sewage sludge on agricultural land ( Latosińska *et al.*, 2021) sewer drop manholes ( Wicke *et al.*, 2021) and gully traps made of cast iron ( Rana *et al.*, 2024) welding operations ( Abdullahi and Sani, 2020), motor vehicles, source of emissions ( Kryshtopa *et al.*, 2019) and second-hand tobacco smoke ( Karatela *et al.*, 2019).

Furthermore, besides point sources (mainly from industrial processes), whereas they can also get into the environment from nonpoint sources (from the atmosphere and from the water, land and biomass already contaminated by a previous heavy metals pollution ( Wang *et al.*, 2022a). When introduced into the environment, heavy metals are exposed to a plethora of environmental processes and are distributed into the atmosphere, water and soil. Once in the environment, heavy metals are either degraded or stored ( Dongre, 2021). The net effect, whether the heavy metals are degraded or accumulated, depends on the duration and the quantity of the release and the difficulty in degrading the compound ( Rahman and Singh, 2020).

As a result of numerous geochemical, mineral residues and deposits, it is very much difficult to categorize heavy metals. Classifying the source of contamination of heavy metals is very difficult because of the various linkage and interconnectivity of a plethora of courses or regions associated with the same origin and nature. Moreso, research has shown different sources following sources based on mechanism of release ( Obasi and Akudinobi, 2020). It is imperative to understand that heavy metals are present in the earth’s crust and are part of the exponential scale of the naturally occurring elements ( Ali and Khan, 2018). The primary source of heavy metals in the environment is the geologic origin of the major and accessory minerals contained in the soil and rocks ( Subasinghe *et al.*, 2022; Hultman and Pollard, 2022).

Heavy metals are naturally occurring elements and are released in diverse ways from rocks and soils ( Bharti and Sharma, 2022). Weathering, tectonic activity, and pan-genetic processes represent a trio of principal mechanisms of heavy metal geological introduction from primary or secondary minerals ( Wu *et al.*, 2021).

Human activities in terms heavy metal release is categorized into two; Essential and non-essential ( Campbell and Gailer, 2016). Essential heavy metals such as copper, zinc, iron, and others are known for their vital role in human health, while non-essential heavy metals such as arsenic, lead, mercury, cadmium, and antimony have been reported to cause a high level of biological toxicity ( Mitra *et al.*, 2022). Human activities (anthropogenic), mainly industrial processes, have been reported to lead to an increase in the release of non-essential heavy metals into the environment at a dazzling rate, leading to an ecosystem crisis ( Mansor *et al.*, 2024). Heavy metal poisoning can lead to a variety of illnesses in humans, some potentially lethal, including blood ( Zahra *et al.*, 2017) and neuromuscular problems ( Toledano, 2020) and many forms of cancer ( Khanniri *et al.*, 2023). Moreso, as human population increases year in year out, heavy metal pollution never decreases instead it increases significantly, leading to potential health hazards as a result of changes in environmental conditions and demographic trends ( Crocetto *et al.*, 2023).

Human beings and the environment is exposed to heavy metals via various sources. Human activities are responsible (anthropogenic activities) for approximately 60% of metal pollution in various terrestrial and aquatic ecosystems throughout the world ( Mitra *et al.*, 2022; Mondal, 2023). The joint precipitation of heavy metals from innate and anthropogenic activities in various environmental spaces has been revealed and reported in various research ( Chen *et al.*, 2021; Verma *et al.*, 2021; Akoto and Anning, 2021; Li *et al.*, 2020). Human activities are the major source of heavy metal pollution globally ( Adnan *et al.*, 2022; Rai *et al.*, 2019). The two most important anthropogenic sources of heavy metals are as follows: (1) Industrial sources: These sources are responsible for the deterioration of air quality within urban and industrial areas ( Roy *et al.*, 2024) (2) Non-point sources: Non-point sources are responsible for the overall deposition of heavy metals within the environment ( Hussain *et al.*, 2023).

### 2.3 Common heavy metals of concern

Over the years, research have shown of that several heavy metals are ubiquitously prevalent in the ecosystem, which are of global concern as a result of to their deleterious effects on human health ( Briffa *et al.*, 2020). Although, many heavy metals, such as copper (Cu), manganese (Mn), iron (Fe), and zinc (Zn), are essential for normal cellular performance, these metals, however, can also be toxic to the human body when present in excess of the normal concentration ( Jomova *et al.*, 2022) Literature has revealed a few popular heavy metals which have shown to cause vicious health effects. Moreso, distinguishing and varying attributes have also been described a plethora of literature ( Meharg and Meharg, 2021; Mishra *et al.*, 2019: Charkiewicz *et al.*, 2023; Baig *et al.*, 2024a; Baig *et al.*, 2024b).

Lead is eccentrically the oldest popularly known and the most surplus heavy metal pollutant/contaminant ( Sable *et al.*, 2024) and it is unsusceptible to corrosion under brassy environmental conditions while being easily transformed into various forms at room temperature ( Rihan *et al.*, 2020). Over the years, lead has been widely used in gasoline (Collin *et al.*, 2022), paint ( Charkiewicz and Backstrand, 2020), water pipelines ( Levin *et al.*, 2021), and batteries ( Kumar *et al.*, 2022), in the production of cooking utensils ( Kuhangana *et al.*, 2024) construction materials ( Dong *et al.*, 2023) and the toy industry ( Yazdanfar *et al.*, 2022). As a result of this, the primary sources of lead exposure have been from the atmosphere ( Wu *et al.*, 2022), water ( Charkiewicz and Backstrand, 2020), food and drinking water ( Gump *et al.*, 2020), contaminated soil and dust ( Stanek *et al.*, 2020) or lead-containing consumer products, especially vintage items ( Guney *et al.*, 2020) and flaking peeling paints ( Afolayan *et al.*, 2021). Also, mercury has been reported in research as a key component of dental amalgam, and arsenic-containing compounds have also been reported being used for chemotherapeutic and insecticidal purposes ( Khatun *et al.*, 2022; Genchi *et al.*, 2020b). Also, mecury and arsenic take part in microbial methylation mechanisms in their inorganic forms which involves the addition of a methyl group to the toxic moiety to form a less toxic, but mainly water-soluble and easily absorbed, methylated compound ( Byeon *et al.*, 2021). Precisely, chronic release of inorganic mercury or other organic moieties in the air to disrupt motor and sensory functions leads to the emergence of *Minamata* disease ( Niede and Benbi, 2022). In the same vein, subtly, arsenic-laden underground water bombards cereals and tubers to develop a mild form of neurotoxicity in contaminated areas due to long-term arsenic exposure ( Sevak and Pushkar, 2024), Methylmercury (Me-Hg) in seafood, particularly in pregnant mothers’ diet, has been implicated to sparks off neurobehavioral symptoms during early development ( Wu *et al.*, 2024), and fish and seafood likely account for 90% of Hg exposure in people residing near rivers and lakes ( Vergara *et al.*, 2024). Furthermore, Cadmium is a highly toxic non-essential element used in electroplating, the television industry, battery production, and coating agents of iron and steel products ( Sable *et al.*, 2024). The major route of human exposure of cadmium is via the ingestion of polluted food, such as rice, shellfish and vegetables ( Zhao *et al.*, 2023) or through inhalation of tobacco smoke ( Genchi *et al.*, 2020b). Alarminly, a huge proportion of cigarettes are haphazardly intermittently irrigated with cadmium fertilizers, which may subsequently release the heavy metal (cadmium) from tobacco during smoking 

### 3.1 Lead

In recent years, widespread human exposure to heavy metals has fascinated increasing public awareness to the probable threat to public health ( Qureshi, 2021). Lead (Pb) has much long been reported by researchers and medical experts to have baleful effects on human health ( Wang *et al.*, 2022b). The major sources of lead exposure are environmental lead pollution ( Raj and Das, 2023). Particularly, there is no safe portal for lead exposure, and the toxicological profile of lead is influenced by the age at exposure, sex ( Gade *et al.*, 2021) and genetic polymorphism ( Sekovanić *et al.*, 2020). Furthermore, lead pollution is an environment-related global health concern, recognized as a public health issue by several global organizations ( WHO, 2023; Muzamil *et al.*, 2024; Słota *et al.*, 2022; Obeng-Gyasi, 2019).

Metallic lead is relatively unreactive under many conditions ( Liu *et al.*, 2021). When heated or subjected to moist air, it can be oxidized and form a variety of inorganic and organic lead compounds, which can further decompose into other lead materials, such as organo-lead and alkyllead ( Nielsen, 2020). Inorganic and organic lead are the two main species linked with detrimental human health effects ( Borah *et al.*, 2020). Due to the long-term presence of lead, in the atmosphere, soil, and water, the exposure of the global population to lead inevitably results in pollution of resources and impacts on the biota ( Mousavi, *et al.*, 2022). Occupational exposures have long been closely associated with painters who use paints containing lead pigments and workers exposed to smoke from burning fuels containing added organic halogenated lead ( Wei *et al.*, 2022; Thangavel *et al.*, 2022). In addition to providing extensive exposure to lead, anthropogenic activities have been reported to trigger environmental spills, such as zinc-lead and copper-silver deposits in the Mississippi valley ( Rosa *et al.*, 2023). The introduction of Tetraethyl lead (TEL) into gasoline supplies also contributed to significant environmental pollution, with large-scale human exposure ( Sarkar, 2020). Lead has also been reported to be released from smelters, recycling centers in municipal areas ( Du *et al.*, 2020) and young ones have been lead-exposed by previously used sources of drinking water ( Olufemi *et al.*, 2022). However, lifestyle choices such as smoking and the use of lead-containing diet fillings have been reported as other means of human exposure to lead. Also research has shown that use of beauty products such as cosmetic products facial creams ( Rico *et al.*, 2023) and surfactants containing lead-based chemicals can result in direct lead toxicity without consuming them ( Abed *et al.*, 2023).

Furthermore, in developing countries, increasing occurrences of lead poisoning have been reported and linked to informal or unregulated electronic waste (e-waste) management practices resulting in severe environmental contamination ( Gollakota *et al.*, 2020) Through various exposure routes (ingestion, inhalation and dermal exposure), lead pollutants threaten human health ( Natasha *et al.*, 2020). Particularly In humans, lead can trigger impacts widely, and these harmful effects are persistent ( Briffa *et al.*, 2020), hence, long-term lead accumulated in the body can increase the gravity of the toxic effects with advancing age. Research have shown that lead exposure to humans can affect the following systems negatively; pulmonary ( Wei *et al.*, 2020), bone ( Boskabady *et al.*, 2022), hepatic and renal systems ( Satarug *et al.*, 2020) neurologic ( Eiró *et al.*, 2021) cognitive and behaviour system ( Shvachiy *et al.*, 2020). Over the years, lead exposure have been reported in a plethora of articles to cause anemia in many individuals ( Wang *et al.*, 2021; Mukisa *et al.*, 2020; Kaneko *et al.*, 2020 ) particularly children being susceptible to the neurotoxic effects ( Brittenham *et al.*, 2023) Some of the weighty consequences of chronic exposure are arthritis ( Fang *et al.*, 2023) and chronic kidney disease that may lead to renal dysfunction ( Balali-Mood *et al.*, 2021). Considering its plausible worrisome effects on the wellbeing of humans and the ecosystem lead exposure is very much a major public health concern globally ( WHO, 2023).

### 3.2 Mercury

Mercury contamination is amongst the most significant and universal pollution problems in the aquatic environment ( Gupta and Yadav, 2024) It primarily occurs in the aquatic environment ( Luo *et al.*, 2020). In industries, huge amounts of effluents containing mercury are discharged as a result of poor industrial operations (pharmaceutical, paint, paper, and other industries), fertilizer industry, landfill leaching, and carbon combustion. Dead zones, otherwise termed as zones of oxygen-depleted water, have been reported to be the repository of huge deposits of inorganic mercury ( AlgarnI *et al.*, 2023; Jonidi Jafari *et al.*, 2020).

The mercury in the environment rapidly makes its entry into aquatic biota. The mercury is consecutively transported to the aquatic food chain via bioaccumulation ( Saidon *et al.*, 2024). Aquatic diet have been reported to contain a huge amount of mercury ( Barone *et al.*, 2021) which puts the concept of food from the aquatic environment in doubt. The widespread presence of mercury contaminated fish in all types of water bodies has grave effects on the global aquatic environment, economy, and public health ( Abhishek *et al.*, 2022). Therefore, it imperative to fathom the coaction between mercury content in fish and the source of water, fish size, and type of species, well-known poisoning effects, extreme intolerance effects, and treatments. Hence, this review will provide an unbiased interpretation of the effects of heavy metal mercury on global health and the aquatic environment.

### 3.3 Cadmium

Cadmium (Cd) is toxic, nonessential, and carcinogenic for humans and animals ( Genchi *et al.*, 2020b). Cadmium exposure to the environment is primarily due to anthropogenic activities ( Zhao *et al.*, 2023; Knoell and Wyatt, 2021; Adil *et al.*, 2020), just like every other heavy metals and not much of natural sources exist. Cadmium have been reported to be present in tobacco products , particularly cigarettes ( Dinh *et al.*, 2021). Also, cadmium is also present in foods, such as shellfish, rice, mushrooms, drinking water, spinach, and other green leafy vegetables ( Genchi *et al.*, 2020b), with rice being one of the major routes of exposure via food. Major sources of cadmium pollution are nonferrous smelters ( Wei *et al.*, 2022), industrial production involving cadmium ( Suhani *et al.*, 2021), incineration of municipal waste and sewage sludges Król *et al.*, 2022). Research have shown that bio- transfer is the common way for the entry of cadmium into the food web ( Peana *et al.*, 2022; Sun *et al.*, 2020) Cadmium is taken up by the plants and also accumulates in the soil ( Sterckeman and Thomine, 2020), it is also easily ignored in the environment for a longer time due to its chemical nature and releases gradually unlike other metals. Also, cadmium takes a longer time to degrade in the environment due to its long biological half-life ( Genchi *et al.*, 2020a).

Human exposure to high-level of cadmium have been reported in a plethora of research to leading to severe damage to the liver, kidneys, and lungs ( Satarug, 2012; Branca *et al.*, 2020; Knoell and Wyatt, 2021; Chandravanshi, *et al.*, 2021; Owonikoko *et al.*, 2023; Farh *et al.*, 2024). Also, lots of animal studies have shown neurotoxic effects as a result of cadmium exposure ( Branca *et al.*, 2020; Zhou *et al.*, 2020; Gade *et al.*, 2021; Ruczaj and Brzóska, 2023; Patel *et al.*, 2021) Clinical symptoms such as headaches ( Söderholm *et al.*, 2020), osteoarthritis ( Frangos and Maret, 2020; Xia *et al.*, 2022) dizziness ( Dumpala *et al.*, 2024), cough ( Li *et al.*, 2020 ), bronchitis ( Ibrahimou *et al.*, 2021), and fever has been reported via research to be associated with cadmium exposure ( Elmas, 2023) Furthermore, plausible bone damages have been reported to be caused by cadmium exposure ( Ma *et al.*, 2022). Also, research as also emphasized in recent years that low-level chronic cadmium may also lead to kidney damage ( Tsai *et al.*, 2021), which primarily focuses on the degree of damage observed in the process of β2-microglobulin excretion and α-glutathione-S-transferase in the urine of human ( Polaka *et al.*, 2023). A recent study has revealed that cadmium-induced renal tubulointerstitial injury might ultimately lead to decreased functionality ( Xu *et al.*, 2021) Also a plethora of research have revealed that environmental that environmental exposure to cadmium also causes endocrine and hormone-disrupting effects ( Rai *et al.*, 2019; Li and Li, 2020; Di Ciaula and Nazarian *et al.*, 2024; Yang *et al.*, 2024). A recent study have also revealed that cadmium exposure in humans through inhalation may induce lung cancer ( Lee and Lee, 2024) and cadmium ingestion through tobacco has been reported to cause oral cancer ( Satir, 2022). In addition, environmental cadmium may be associated with primary liver cancer ( Cirovic and Satarug, 2024) Research has also revealed that children exposed to cadmium via dietary intake exhibit lower intelligence than those not exposed ( Kampouri *et al.*, 2024).

### 3.4 Arsenic

Arsenic is a metalloid which is naturally present lithosphere ( Meharg and Meharg, 2021). It enters the environment either due to anthropogenic usage or through removal from iron, manganese, and aluminum oxides ( Raju, 2022). Several anthropogenic sources, such as mining activities, smelting, and burning of fossil fuels, release arsenic into the environment ( Kar, 2022). Chronic or acute exposure to arsenic have been reported to cause debilitating health effects ( Thankachan *et al.*, 2023 Muzaffar *et al.*, 2023; Rehman *et al.*, 2020). Organo-arsenic compounds dominate arsenic exposure, but these compounds have been reported to transform into inorganic forms and hence more toxic forms in the body ( Valskys *et al.*, 2022). Groundwater is the primary source of arsenic exposure ( Monteiro De Oliveira *et al.*, 2021), about 21 countries have been found to contain arsenic-ridden drinking water ( WHO, 2022).

Chronic exposure to arsenic-rich drinking water may lead to the development of skin lesions ( Rajiv *et al.*, 2023; WHO, 2022), internal cancers of the blood vessels ( Rahaman *et al.*, 2021) or urinary bladders among others ( Jaafarzadeh *et al.*, 2023). This phenomenon have been reflected in a plethora of studies ( Mayer and Goldman, 2016; Kumar and Ghosh, 2019; Rahaman *et al.*, 2021; Kumar *et al.*, 2022; Rehman *et al.*, 2020; Chikkanna *et al.*, 2019). Cadmium has been implicated in several biological pathways to cause carcinogenesis ( Peana *et al.,* 2022; Zhu and Costa, 2020; Cui *et al.*, 2021; Luparello, 2021) including oxidative stress ( Branca *et al.*, 2020). mitochondrial DNA damage and apoptosis ( Mohamed, 2022) impairment of DNA methylation ( Genchi *et al.*, 2020b), and changes in methyltransferases ( Sun *et al.*, 2021). Moreso, It is acute arsenic exposure can lead to systemic poisoning and ultimately death ( Ganie *et al.*, 2024). A single lethal dose of arsenic compounds ranges between 100 to 200 mg ( Chen *et al.*, 2021). Symptoms of acute poisoning include diarrhea, poor appetite, extreme tiredness, sores on the skin, numbness, muscle cramps and hair loss ( Rehberg and Rehberg, 2024) However, acute poisonings from waterborne arsenic exposure are uncommon ( Roy and Edwards, 2022). Long-term skin exposure may cause skin changes, such as darkening of the skin ( Passeron *et al.*, 2020) and the appearance of small “corns” or “warts” on the palms soles, and body ( Hamza *et al.*, 2022). Also, research studies have revealed plausible effects of arsenic exposure on reproductive health and impaired fetal brain development ( Dutta *et al.*, 2022).

## 4. Routes of human exposure and mechanisms of heavy metal toxicity

### 4.1 Routes of human exposure to heavy metals

The avenues through which humans come into contact with heavy metals vary greatly between different contaminants and among different populations. Ingestion represents the main route of exposure for most human population groups ( Kabir *et al.*, 2022). Affected individuals generally include those living close to contaminated areas , those accidentally exposed to pollutants and occupationally-exposed individuals . Inhalation ( Briffa *et al.*, 2020) and, to a lesser extent, dermal contact may also represent more relevant exposure routes for special populations, such as workers engaged in mining smelting ( Xu *et al.*, 2021; Li *et al.*, 2020) or in the production and application of herbicides, pesticides, and fertilizers . Human exposure to heavy metals includes, at least, ten different interconnected exposure pathways. These pathways include affected food and drinking water resources and lead to the wide distribution of hazardous heavy metals in the tissues, organs, and fluids of the human body ( Zhao *et al.*, 2022). Taken together, human intake of heavy metals is driven by lifestyle local environment ( Mitra *et al.*, 2022) occupation ( Baig *et al.*, 2024b), nutrition and the occurrence and concentration of heavy metals in affected air, water, and soil resources ( Fu and Xi, 2020).

Ingestion represents the major pathway of human exposure to heavy metals . A number of food sources contain considerable levels of heavy metals, such as crops grown in heavy metals-contaminated soils ( Bwatanglang *et al.*, 2022) fish from mercury-contaminated waters and beverages containing lead carried by deteriorated distribution systems. Human dietary intake typically ranges from approximately 0.1 to 1.0 g/day, with higher intake levels observed in diets rich in seafood and other wild foods. Overall, high daily intakes and the evidence from research suggest that moderate to high levels of heavy metal residues in food and water can trigger significant toxicological effects in humans ( Ahmad *et al.*, 2021).

**a. Ingestion**

The major medium for human exposure to toxic metals is ingestion ( Dippong *et al.*, 2024). This is facilitated by the consumption of micro- or macro-nutrients and subsequent accumulation of metals in human food, water, air, or soil ( Kara *et al.*, 2024). An acute part usually comes from water used for gardening or agriculture ( Munir *et al.*, 2021). Stationary plants take up toxic metals from the soil and enrich the water table, this water is used for cultivating terrestrial and aqueous food as well ( Zheng *et al.*, 2023; Chaturvedi *et al.*, 2021; Wicke *et al.*, 2021). Research have shown that children are particularly susceptible to soil contamination due to hand-to-mouth activities and due to their low body mass ( Karatela *et al.*, 2020) and potential for higher ingestion of air, water, and food in comparison to adults on a body weight basis ( Frings *et al.*, 2024; Ahmad *et al.*, 2021). During pregnancy, the narrow teenaged pelvis tends to concentrate nutrients and lead into mothers’ bone thickness ( Haeusler et al., 2021) calcium is rapidly pulled out during menopause. The lead stored in the bones may cause fetal wastage, fertility decrements, and low birth weight (Collin *et al.*, 2022). Exposure via this route may result in poor breathing and hence directly absorbed through cell membranes of the lung ( Khoshakhlagh *et al.*, 2024). Water can also be very high in toxic metals and hence be detrimental to the consumer ( Briffa *et al.*, 2020). Food grown near or processed with such commodities from contaminated water will also result in toxic ingestion ( Yüksel *et al.*, 2023 ). The taste of water is important to determine when water becomes unfit for human consumption; therefore, heavy metals in solution or in a state of suspension can be ingested through water as drinking water or through food and is vital to prevent water and food pollution ( Mazinder Baruah and Singh, 2022; Sonone *et al.*, 2020). Fish rearing in polluted soil, nitrogenized ponds take up metals via food and gill; hence fish as a food can affect the public and should not be cooked and fed to children ( Porretti *et al.*, 2022). Fish is a good indicator for bioaccumulation of metals ( Sheikhzadeh and Hamidian, 2021; Pironti *et al.*, 2021).

**b. Inhalation**

Humans are predominantly exposed to toxic heavy metals from a plethora of industries in the surroundings ( Singh *et al.*, 2023). Inhalation happens as a result of direct exposure; as a result, it is one of the most hazardous routes ( Yasmeen and Hafeez, 2023; Sonwani *et al.*, 2022; Kurt and Basaran, 2020). The heavy metals are present both in particles and in fumes ( Hedberg *et al.*, 2021; Sonwani *et al.*, 2021). Inhalation of particles is more common, although heavy metals are excreted from the system as they are insoluble in water. Heavy metal inhalation have been reported to cause a plethora of disorders that exert their effects on numerous body systems ( Fulke *et al.*, 2024). Even if the systemic manifestations are not noticeable at the outset, the disease will ultimately harm other systems, ( Abd Elnabi *et al.*, 2023) particularly the central nervous system . Approximately 95% of inhaled lead particles are 1-2 μm in diameter and therefore do not eliminate easily and persist in the body for a long time. Respiratory disturbances can be induced by long-term or higher-lead exposure ( Dumková *et al.*, 2020). Airborne free cadmium have shown to produce hazardous effects by the respiratory system ( Bhattacharyya *et al.*, 2023); conversely, its oxide and chloride have been reported to have no effect ( Genchi *et al.*, 2020a). Moreso, exposure to a higher level of cadmium have been reported via literature to cause inflammation and emphysema ( Wang *et al.*, 2024). Also, there is strong evidence from literature that heavy metal have impacted workers; revealing nickel refinery workers have been reported to have more nasal and nasal-chronic disorders than non-impacted workers ( Kurt and Basaran, 2020; Syurin and Vinnikov, 2022). Cadmium sulfate and smoker’s cadmium have been revealed to be deleterious to the upper respiratory pathways ( Genchi *et al.*, 2020b).

**c. Dermal contact**

Heavy metals are a growing threat to general public health and exhibit known toxic impacts on humans and the environment ( Mitra *et al.*, 2022) Nowadays, heavy metals are scattered in every nook and cranny of the environment ( Adeola, 2020; Kumar *et al.*, 2023). The circulation of heavy metals occurs through the air, water, and food cycle, with plants representing the primary source ( Sonone *et al.*, 2020). Nonetheless exposure to heavy metals can also be via the skin. The dermal surface area in adults varies from 1.5 to 2 m ^2^, whereas in children, the ratio of skin surface area to body weight is higher ( Zhao *et al.*, 2023). Hence, children are at a higher risk of exposure to heavy metals in the environment compared to adults ( Zheng *et al.*, 2023). Even dust on the floor of houses and soil can cause health problems when inhaled or ingested with food ( Roy *et al.*, 2024). The uptake of heavy metals, either through respiration or via the skin, now creates debilitating health hazards ( Edo *et al.*, 2024; Passeron *et al.*, 2020). To determine the significance of dermal contact as a route of human exposure to toxic metals, it is imperative to fathom that wherever required, metal ions must be mobilized from the surface of a contaminant particle that contacts human skin. However, toxic metals are normally present in an oxidized or insoluble state in natural environmental particles ( Saravanan *et al.*, 2022).

### 4.2 Mechanisms of heavy metal toxicity

Heavy metals exert their toxic effects in a highly complex process with thorough mechanisms which are yet to be understood ( Mitra *et al.*, 2022; Engwa *et al.*, 2019). However, several direct mechanisms have been proven to be involved in exerting the deleterious effects of heavy metals including oxidative stress DNA damage ( Fu and XI, 2020), membrane damage quenching of in vivo antioxidants ( Pisoschi *et al.*, 2021), protein dysfunction and enzyme inactivation ( Balali-Mood *et al.*, 2021),

In an effort to elucidate the mechanisms of metal toxicity, Shi *et al.* (2004) reported that via oxidative stress, heavy metals were able to form complexes with biological molecules such as lipids, proteins, and DNA, which lead to cell injury by generating or regulating the content of reactive oxygen species (ROS) effects ( Acharya, 2024; Fasae and Abolaji, 2022). Reduced antioxidant status effects and dysregulation of antioxidant enzymes have been reported to decrease antioxidant status effects and allow formation of free radicals, thus cumulative body injury ( García-Sánchez *et al.*, 2020). Metallic oxides are able to reduce cytochromes and to generate active oxygen species this can further result in cellular levels of adsorption and the resultant genomic deoxyribonucleic acid (DNA) methylation ( Mitra *et al.*, 2022). All of the aforementioned mechanisms may gradually results into carcinogenesis. A review by Adil *et al.* (2020) heavy metals in various cells were able to induce changes in signal transduction pathways, thus alterations in gene expression, DNA repair organelles, cause inflammation, and genotoxic stress. All these changes ultimately lead to carcinogenesis. One highly prominent DNA modifying mechanism that is thought to occur is the generation of ROS. In addition to ROS generation, metals have also been found to have a direct effect on DNA via the formation of metal-DNA adducts where a single base pair is displaced and metal ions then sit in the space created ( Marchi, 2023).

**a. Oxidative stress**

The human body has a conglomerate of elements such as heavy metals that perform numerous functions in the body ( Mitra *et al.*, 2022: Some metals are essential for the homeostasis of the organisms (REF). while some are toxic when they overly interact and get supplemented in the cells (REF). A plethora of heavy metals such as lead, mercury, arsenic, and cadmium have no specified physiological function ( Balali-Mood *et al.*, 2021). Above that, they disrupt numerous enzymatic processes happening inside the body ( Muzaffar *et al.*, 2023). The generation of reactive oxygen species (ROS) is noticeable by metals, concerning oxidative stress that ultimately leads to imbalances between the body’s antioxidant defense mechanisms and prooxidants ( Afzal *et al.*, 2023). In recent years, extensive heavy metal poisoning cases have been reported in a plethora of research ( Guo *et al.*, 2020; Hu *et al.*, 2020; Obasi and Akudinobi, 2020). Hence, it has become an imperative area of research to investigate the source and remediation of heavy metals. Oxidative stress is an unpleasant condition created by the buildup of free radicals or any mismatch between the production of free radicals and application of antioxidants ( Marchi 2023; Martemucci *et al.*, 2022). Many pathological and psychological disorders have been reported to have been caused by oxidative stress in the human body ( Kowalczyk *et al.*, 2021). It can further spur up numerous acute or chronic diseases ( Wood *et al.*, 2021). If the balance between the formation of free radicals and the ability of cells to survive against a redox any of an abnormality in the production of antioxidants surpasses the free radicals, oxidative stress will occur ( Chaudhary *et al.*, 2023). In the cells of humans oxidative stress gives rise to the formation of free radical products that are life-threatening to the physiology of the cell ( Zahra *et al.*, 2017). Heavy metal toxicity can directly be linked with oxidative stress, either by redox-cycling or by deactivation of antioxidant systems ( Nowicka, 2022). Antioxidants act as a natural defense system and neutralize excessive production of oxidants in the body due to any plausible agents ( Pisoschi *et al.*, 2021).

**b. DNA damage**

Over the years, several studies, including in vitro and in vivo studies, have shown the direct and indirect genotoxic and cytogenetic effects of several metals ( Sánchez-Alarcón *et al.*, 2021). Damage of DNA can be caused by metals such as Ni, NiO, and NiSO4 ( Genchi *et al.*, 2020a). DNA damage have been investigated in irradiated and non-irradiated cells/nickel-containing solution, revealing he results of this study showed that DNA strand breaks, induced by nickel-titanium (NiTi) and pure nickel under the presence of ultraviolet light caused the result of the synergy between the UV light and the nickel ( Umaña *et al.*, 2024). The mechanism(s) leading to nicking of DNA by nickel is not yet fully understood in the scientific world understood, but studies have shown that nickel binding to chromatin can affect the structure of the DNA and greatly increase susceptibility to strand scission by stripping the histone proteins from the chromatin ( Kasprzak, 2023; Sekovanić *et al.*, 2020).

Heavy metal-induced DNA damage is a serious risk factor for many human health issues, including cancer ( Paithankar *et al.*, 2021), aging ( Vielee and Wise JP Jr *.*, 2023), and neurodegenerative diseases ( Stoccoro and Coppedè, 2024). The damaging effect of exposure to metal ions and other nonessential metals on DNA has been investigated in many in vitro and in vivo studies ( Ren *et al.*, 2024; Kasprzak, 2023; Fasae and Abolaji, 2022; Slobodian *et al.*, 2021; Peana *et al.*, 2021). The negative effects of heavy metals on genetic material occur mainly at four major levels: cytotoxic, genotoxic, mutagenic, and carcinogenic, depending on the exposure dose, duration, and concentration ( Dutta and Ruden, 2024; Stoccoro and Coppedè, 2024; Mitra *et al.*, 2022).

**c. Protein dysfunction**

Changes in protein expression and phosphorylation have been reported in several tissues after exposure to heavy metals ( Mondal, 2023; Mansoor *et al.*, 2023; Renu *et al.*, 2021; Fu and Xi *et al.*, 2020). The human body has several classes of proteins, including enzymes with well-conserved active sites, transmembrane proteins, signal transduction proteins, or transporters that heavy metals may bind to, disrupting their function ( Riziotis and Thornton, 2022). Consequently, heavy metal-binding proteins may not be released from the transporter or receptor, forming a new entity that may continuously send signal transduction, resulting in persistent acute or chronic contamination, e.g. lead intoxication , Furthermore, some proteins have been reported to be sensitive to heavy metals ( Pillai *et al.*, 2020). Although, the effect that heavy metals have on proteins is not always very much clear but there may be a catalytic role for the heavy metals, for example Cd have been reported to collapse the structure of the hemoglobin tetramer, leading to the release of free alpha plus Cd and beta chains ( Pillai *et al.*, 2020).

Furthermore, cadmium have been implicated to affect the enzymatic function of several proteins, including kinases, transcription factors, and metalloprotease ( Mondal, 2023). Chronic exposure of patients (humans) to cadmium (i.e., Cd-induced itai-itai disease) has been reported by medical experts ( Dutta *et al.*, 2022; Sakurai *et al.*, 2023) and sporadically accelerating breakdown of postmenopausal women bone by inhibiting the function of matrix metalloproteases, which stimulates bone formation and thus exacerbates the hypercalciuretic effect on the body ( Ciosek *et al.*, 2023 Rastgar *et al.*, 2022). Also, lead exposure have shown to cause causes amino acid substitution and increased expression of heavy metal-responsive proteins, accompanied by the induction of heme oxygenase-1 ( Kapoor *et al.*, 2021; Haeusler *et al.* 2021).

## 5. Health effects of heavy metal exposure and case studies

Throughout the regions of the world, humans are habitually exposed to various toxic metals via water, food, and air ( Parui *et al.*, 2024). In the Homo sapiens species these metals are known to bioaccumulate and biomagnify thereby leading to injury in a plethora of body systems ( Parida and Patel 2023; Shah and Kumar, 2022; Semwal *et al.*, 2022). High level of heavy metal in the blood may result into a negative cardiovascular effects with individuals showing signs like increase in heart rate ( Mitra *et al.*, 2022) and arterial stiffness ( Wan *et al.*, 2023). Some heavy metals that can cross the blood-brain barrier induce neurotoxic effects such as neuropathy and cognitive dysfunction or encephalopathy ( Singh and Sharma, 2021; Baig *et al.*, 2024a). Other non-neurotoxic symptoms, such as gastric erosion and vomiting, increase the mortality risk for poisonings and septicemia when exposed to high levels ( Parida and Patel 2023; Benhalima *et al.*, 2023).

Long-term exposure to lead and cadmium is the most common causes of chronic kidney disease and is significantly associated with decreased estimated glomerular filtration rate, consistent with the underlying pathology of chronic kidney disease ( Satarug *et al.*, 2020). In pregnant mothers, babies, research has shown that at a level of mercury in the form of methylmercury of 1 ppm can result into numerous inappropriate effects on brain development ( Fujimura and Usuki, 2022). Also, research has shown via animal experimental studies that low accumulation of heavy metals in tissues can cause adverse effects on the reproductive system such as sperm quality ( Heidari *et al.*, 2021) hormone levels, mutations, sperm necrosis, fertilization capacity and pregnancy . However, some cases of human death have been reported via due to oral exposure to heavy metals ( Street *et al.*, 2024; Fu and Xi, 2020; Satarug *et al.*, 2020 M Balali-Mood *et al.*, 2021). Besides all of these aforementioned effects, heavy metals have also been reported to propagate tumorigenesis, thereby leading to cancer ( Briffa *et al.*, 2020). A detailed understanding of the mechanisms of improvement and toxicity of heavy metals will help to provide an improved specialized approach.

Taken together, a plethora of studies have described and shown the toxic effects on the nervous, cardiovascular and reproductive systems after exposure to heavy metals, and also suggest that the administration of heavy metals in medical applications should be viewed by the accumulating body of evidence as a potentially hazardous route ( Mitra *et al.*, 2022; Taslima *et al.*, 2022). In addition, these heavy metal-induced diseases have deleterious impact on the quality of life of individuals ( Briffa *et al.*, 2020).

### 5.1 Neurological effects

The deleterious effects observed by the effects of heavy metals may be in the form of neurodevelopmental disorders like autism ( Błażewicz and Grabrucker, 2022) and attention deficit/hyperactivity disorders ( Gu *et al.*, 2024). Medical experts have reported cognitive impairments in patients and seizures as a result of heavy metal contamination (Singh and Sharma). Along with these, changes in the level of some neurotransmitters like acetylcholine, noradrenaline, and dopamine in humans as a result of heavy metal accumulation ( Pyatha *et al.*, 2023). Hence, suggesting that neurotoxicity has a huge health effect which should be considered at heavy metal-contaminated sites ( Baig *et al.*, 2024b). Also, Heavy metals are known to act as neurotoxicants to all age groups of humans ( Gade *et al.*, 2021). Exposure to heavy metals during pregnancy have been reported to result into various neurodevelopmental disorders in children ( Ijomone *et al.*, 2020; Heng *et al.*, 2022; Ding *et al.*, 2023; Farmani *et al.*, 2024; Nehzomi and Shirani, 2024). The cognitive functions of exposed children have been reported to be pathetic ( Ding *et al.*, 2023). Lead has been implicated to cause as a result of exposure in children ( Parithathvi *et al.*, 2024). About 1 in 100 children has autism of people have been diagnosed with autism ( Nehzomi and Shirani, 2024; WHO, 2023). A study shows that children with autism spectrum disorder have lifted harbors of various heavy metals-small molecular weight proteins such as zinc and copper ( Błażewicz and Grabrucker, 2022). In recent literature, mercury, lead, arsenic and cadmium have emerged as the two most common heavy metals linked to attention deficit hyper activity disorder (ADHD) in recent literature ( Dutta *et al.*, 2022). The deficiency in zinc in the body of the individual is responsible. Noradrenergic and dopaminergic dysfunction have also been reported in heavy metal neurotoxicity ( Medda *et al.*, 2020). Other forms of toxicity reported via literature in terms of prolonged exposure, is a decrease in the level of acetylcholine, which leads to cognitive and behavioral alterations ( Althobaiti, 2024). Apart from all of these, effects like Parkinsonism, depression and anxiety have also been reported in recent studies ( Mitra *et al.*, 2022; Vellingiri *et al.*, 2022; Baig *et al.*, 2024a; Tizabi *et al.*, 2024). The presence of genotoxicity on neurological systems of patients on exposure to heavy metals has also been reported via literature ( Stoccoro and Coppedè, 2024). Taken together, neurotoxicity is basically one of the important endpoints for site assessment and clean-up goals, and immediate action should be taken to prevent heavy metal exposure.

### 5.2 Cardiovascular effects

Animal studies of heavy metals have revealed cardiovascular effects ( Sonone *et al.*, 2020; Mitra *et al.*, 2022). This strongly indicates an association between heavy metals and the development of hypertension or hypertension-induced end-organ damage. Cadmium is the most widely studied heavy metal with respect to cardiovascular toxicity ( Garai *et al.*, 2021). Experimental studies suggest that Cd in a general population is associated with atherosclerosis or peripheral arterial disease ( Barregard *et al.*, 2021). Also, lead has been implicated in numerous processes associated with cellular stimulation leading to atherosclerotic plaque formation ( Libby, 2021), as well as affecting the integrity of blood vessel walls through neurotoxic and cardiotoxic effects ( Rajpoot *et al.*, 2024). The mechanism that shows the effects of another heavy metal, arsenic, on cardiovascular health is yet to be understood. In a study that included some Bangladesh’s rural community members as well as individuals from a more general (urban as well as rural) health center, the authors reported an exposure-response relation between arthritis and heavy metal exposure, particularly for inorganic arsenic ( Choi *et al.*, 2011). Taken together, evidence suggests that agricultural tasks can inadvertently lead to the uptake of high levels of heavy metals and that these heavy metals may act in a discriminatory nature, targeting the meninges and/or dopaminergic system, culminating in Parkinson’s disease.

### 5.3 Reproductive effects

Reproductive structures are very much imperative not only for existence of an individual but also for growth, enlargement and multiplication ( Massányi *et al.*, 2020). The response of reproductive organs to toxic substances differs from that of other target organs, and they may serve as an ideal measurement for the deleterious effects of environmental contamination on animal and human health ( Massányi *et al.*, 2020). Heavy metal exposure can cause a plethora of deleterious effects on the reproductive region of human health ( Fulke *et al.*, 2024). Heavy metals can cause sperm DNA damage and lower sperm quality, thus affecting male fertility ( López-Botella *et al.*, 2021) Heavy metals can also disturb sex steroid hormone levels and types of semen, resulting in subfertility ( Bhardwaj *et al.*, 2021). On the other hand, lead, cadmium, and mercury can damage the ovaries of females who are then further at the risk of giving birth to anatomically or neurologically malformed offspring because of heavy metal-induced prenatal effects ( Yan *et al.*, 2023). During the entire process of maturation of sperm, the cells are exposed to generated reactive oxygen species. This excessive exposure can lead to oxidative stress resulting in sperm DNA damage ( Gautam *et al.*, 2024), which can be one of the reasons for making sperm immobile. Lead has been reported in literature to disrupt sex steroid homeostasis in human circulation ( Kasten-Jolly and Lawrence, 2017). Lower levels of luteinizing hormone, progesterone, and 17OH-progesterone have been linked with an increase in cadmium accumulation in testes and seminal plasma of infertile and asthenospermic men ( Obasi *et al.*, 2022). Also, research as shown that once copper gets absorbed into the systemic circulation, it temporarily disrupts spermatozoa production, maturation, and hormonal homeostasis ( Bhardwaj *et al.*, 2021). Meta-analysis evidence has revealed that the epididymis is very much sensitive to heavy metal contamination ( Machado-Neves, 2022). Furthermore, lead and cadmium are endocrine disruptors that harm the male and female reproductive system, thereby disturbing the fertility of humans. Cadmium for example was reported by Machado-Neves (2022) to be deleterious to the epididymis, leading reduction in the weight of the epidermis and reducing the number of sperms. Moreso, aluminum has also been implicated cause reduction in blood level of FSH and LH ( Ojoghoro *et al.*, 2021), therefore affecting male fertility ( Ali *et al.*, 2024; Di Ciaula and Portincasa, 2021). Similarly in females, heavy metals can cause negative effects in the female reproductive tracts, a recent study by Tian *et al.* (2024) revealed that lead is highly linked to diminished ovarian reserve at during the reproductive age of females. A plethora of research over the years have implicated cadmium in the reduction of ovaries in females ( Nna *et al.*, 2017; Nasiadek *et al.*, 2019; Massányi *et al.*, 2020; Ruslee *et al.*, 2020). Exposure to lead and transfer of lead across the placenta may cause miscarriage in females and subfertility ( Dutta *et al.*, 2022). An increased spontaneous abortion rate as a result of lead poisoning has also been reported in animals ( Mosaad *et al.*, 2024). Therefore, essential to assess the reproductive health of females areas in the environment likely to be contaminated with heavy metals.

### 5.4 Itai-Itai disease

Itai-itai disease is the most popular case of cadmium (Cd) pollution reported in Japan ( Sasaki *et al.*, 2024). The term *"itai-itai"* is from a Japanese word which means “it hurts, it hurts” in English vocabulary ( Kaji, 2015). Around 1500 people had severe symptoms such as liver damage, osteomalacia, and bone deformities during the initial phase of the outbreak . Mostly women were the victims, and the male to female ratio of the patients was 1:2 or 1:3 ( Kasuya, 2000) Furthermore, The damage it caused in the Jinzu River basin (Japan) and surrounding areas in Toyama Prefecture was recorded by Kozukue as early as 1981. It was reported that the cause of the itai-itai disease was huge ingestion of rice. Much higher cadmium levels were found in the rice which was consumed by patients than by people living in the non-polluted areas ( Kasuya, 2000).

The main clinical symptoms were osteomalacia, renal dysfunctions, and pathological fractures. It was also reported that there were single cases of lung and prostatic cancers among the patients ( Zhang *et al.*, 2023). The cases of chemical-induced itai-itai patients, especially, showed severe osteomalacia manifested in similar deformities of the elbow, femoral neck, and knee.

## 6. Case studies of heavy metal pollution in Nigeria

### 6.1 A case study at Ondo Town, Nigeria

In a recent study by Orimisan *et al.* (2024) investigation of precisely five heavy metals (copper, lead, and nickel iron, cadmium,) were scrutinized in two vegetables ( *Talinum triangulare* and *Chromolaena odorata*) at two dumpsites in Ondo town, Nigeria. A dumpsite located between Ondo Town and Okeigbo was represented as site A and another dumpsite located at Okelaje-Ondo road was represented as Site B. Surprisingly the heavy metal levels identified in and vegetables were below WHO threshold limit with the exception of Cadmium in the control site except for cadmium in site A which was slightly higher. The heavy metals concentration examined in this investigation followed the sequence Fe > Cu > Cd > Ni > Pb for soil from locations A, and Fe > Pb > Cu > Ni > Cd for soil from sites B The dumpsites and the tested two vegetables revealed elevated levels of the heavy metals in comparison to the control area, suggesting a gradual accumulation of these metals in these vegetables. Although the concentration of heavy metals detected in the vegetables in these areas in Ondo town had minimal levels of the selected heavy metals tested, but the fact that they were present in the vegetables is a huge problem, hence may bio accumulate gradually, integrate into the food chain causing chronic debilitating health hazards.

### 6.2 Case study of Gashua town, Bade Local Government area Yobe

A study by Suleiman *et al.* (2021) revealed heavy metal contamination in drinking water at six wards in Bade local government area, Yobe, Nigeria, which was as a turnout of pollution in the environment, ultimately affecting the drinking water in Gahua town Gombe negatively. The level of concentration of the following heavy metals; Iron, Manganese, Cadmium, Cobalt, Copper, Mercury and Nickel in boreholes across six wards the Gashua metropolis were assayed. The wards are as follows; Lawan Musa, Sabon Gari, Sarkin Hausawa Katuzu, Lawan Fannami, and Zango Wards. Water samples collected from eighteen sampling point across six urban wards showed no significant difference in terms of heavy metal contamination. Albeit, Suleiman *et al.* (2021) suggested that water from some ward were not suitable for drinking because of the huge build up some of the heavy metals that may cause kidney stones or renal failure.

The mean concentration from the study by Suleiman *et al.* (2021) for Cadmium showed higher levels in Lawan Fannami, Zango and Lawan Musa wards. Sabon Gari and Lawan Musa revealed higher values of Copper (Cu). Katuzu and Lawan Fannami have higher value of iron concentration that is higher than the acceptable standard, suggesting if precautionary measures are not taken it may lead to hideous diseases which can lead death. Alarmingly, all the six wards from this study have higher value of Lead (Pb) that is higher than the accepted standard using USEPA standard of 0.015 mg/kg. The continuous accumulation of this element may lead to serious health problem such as renal failure and kidney stones. Katuzu ward revealed higher concentration of Manganese (Mn) of 0.27, which is higher than the accepted standard. Zango, Sarkin Hausawa. The mean concentration for Mercury (Hg) shows that they are within acceptable value with no trace in Sabon Gari ward while a higher value in Lawan Fannami ward.

Taken together, this case study suggests a heavy metal contamination is of great concern in Nigeria owing to the fact that there is the presence of very much common heavy metals that may be injurious to the health and can cause serious havoc to the body of human and may even results into death.

### 6.3 Case at the Great Kwa River, Calabar

A recent study by Nneoyi-Egbe (2024) on heavy metal contamination on shrimps and water at the great Kwa river, Calabar Cross River, Nigeria revealed pollution of both the shrimps and water from the Great Kwa River by a plethora of heavy metals, which in turn poses a health risk to aquatic ecosystem and humans. Amongst the heavy metals studied in this research in Calabar, Copper had the highest concentrations in shrimps (33.17 ± 0.79 ppm) and chromium water (27.68 ± 0.34ppm). The heavy metals were found in higher concentrations in the shrimp than in water, except for manganese which had a significantly (p<0.05) higher concentration in water (15.05 ± 0.67ppm) as against 14.02 ± 0.93ppm in shrimp).

The results of the research indicate significant Lead, Cadmium, Nickel, Chromium, Copper and Manganese content in shrimps and water from the Great Kwa River. It is a matter of great concern for both the environment and public health. Heavy metals are known to be toxic and can cause a range of health problems in humans, including neurological disorders, cancer, and kidney damage. Lead, Cadmium, Nickel and Chromium are particularly dangerous because they can accumulate in the body over time and cause chronic health effects. Moreover exposure to these heavy metals is associated with an increased risk of cardiovascular diseases, hypertension, and impaired cognitive function, neurological and behavioral changes, leading to decreased mobility, altered swimming behavior, and reduced foraging abilities. Moreover, lead can accumulate in the tissues of shrimps over time, leading to chronic exposure and bioaccumulation. This can result in bio magnification, a process where the concentration of lead increases as it moves up the food chain, ultimately affecting humans who consume contaminated seafood. Lead contamination in water can also have significant environmental and public health impacts. High levels of lead in water can cause a range of health problems in humans, including developmental delays in children, decreased intelligence quotient, and an increased risk of cardiovascular disease in adults. Taken together, for this case study, heavy metal concentration was present more in Fish than water at the great Kwa river, and this may cause deleterious effect to the humans consuming shrimps and periwinkle in that area.

### 6.4 Case of lead poisoning in Zamfara state

Following a report on 6th November 2009, the Zamfara State Ministry of Health confirmed an unprecedented outbreak of child mortality in eight of the fourteen local government areas of the Shinkafi and Bakura communities in Zamfara State, Northern Nigeria. Affected communities were predominantly peasant farmers. ( Augusto *et al.*, 2021). The mortality affected 34.6% of the population, with a case fatality rate of 30.5%. Over 52% of those who died were children less than five years old ( Augusto *et al.*, 2021). It was further reported that the cause of the deaths was that the children had excessively high levels of lead in their blood. Four to five months later, two sister communities in the Gusau local government area of the state became afflicted as well. These two affected communities are situated away from the six contaminated villages in Bukuyyum and Anka local government areas of Zamfara State ( Abdullahi and Lasisi, 2024).

### 6.5 Case of Ogoni, River state

The Ogoni land is an oil-rich region located in the Niger Delta region of Nigeria. The land and its ethnic group, the Ogoni people, shot to worldwide attention in 1992 when the Nigerian military dictator suppressed a demonstration by the Ogoni people. The Ogoni land can best be described as the treasure or scrap of Nigeria ( Amosu and Adeosun, 2021). The authorities ordered the arrest and subsequently the trial and execution of nine of the movement’s leaders, including the most prominent and their offense was murder ( Okpebenyo *et al.*, 2023). They had been convicted on what are generally considered to be false testimony and mishandled evidence. The movement fought against the disregard of environmental best practices by oil prospecting companies that held sway in the Ogoni land ( Okpebenyo *et al.*, 2023). Companies developing the area were particularly criticized. Areas were inadequately treated after drilling had been completed, and the local people received little or no benefits from oil prospecting ( Sam *et al.*, 2022).

Many lives have been lost indirectly and directly from the activities of the oil and gas explorers in Ogoni land and other inhabitants near these wells and rigs ( Nwoma and Anyika, 2024). The entire ecological system of Ogoni land is severely compromised, and the future of the people of Ogoni is shaky. Cases of massive standing surface oil pollution are very visible in numerous places in the area. Ogoni oil has gained a bad reputation among heavy metal users. In some cases, just the mention of the origin of the crude oil is enough to lose a sale or a customer ( Ben, 2022). Any breakdown of the process control in the industry processing Ogoni crude oil must result in a heavy metal disaster of of world proportions. The Ogoni land can best be described as the treasure or scrap of Nigeria ( Idialu, 2021).

### 6.6 Case of Zamfara gold mine cases and the Itakpe Iron ore mine

Three major goldmines attributed to two distinct lead poisoning epidemics of extraordinary scale and remarkable geological background were recorded in Nigeria in recent history. The first, recorded in Bagega, was in 2010 ( Jamilu, 2023). In the aftermath of an episode of acute fatal childhood lead poisoning, with more than 400 elevated blood lead levels. What can be described as the second major goldmine activity occurred in Anka Local Government of Zamfara in 2020, with a similarity to the Bagega contamination ( Abu Khatita, 2024). Recorded cases of children with elevated blood lead levels have ranged from 24.3% to 60%; over 350 children were reported to have died, and in both of these cases, ( Akinwumi *et al.*, 2023; Ezechukwu, 2023; Wilde *et al.*, 2024) the mining involved grinding and milling of lead-rich ore, exposures from dust inhalation, and other pathways of the soil and water compartments ( Mahdi *et al.*, 2023). Yet a third contamination with no epidemic status and of considerably fewer numbers was recorded in the Tsafe area in 2020, and the 2020 official status alone still pins both it and the 2020 exceedance incidences as being among the largest recorded in research that examined this throughout the world ( Kasongo *et al.*, 2024). A characteristic that has generally defined gold mining in Nigeria is that the mining and grinding of ore releases airborne dust that contains lead, cadmium, mercury, cyanide, and/or other substances used in the process ( Escobedo-Monge *et al.*, 2024).

Another incident of heavy metal contamination with conditions similar to the case described above occurred at the Itakpe iron ore mine in Nigeria in 1985 ( Akande *et al.*, 2020). Geologically, there are suspicions that manganese was present in heavy metal concentrations, both as an oxide and as a carbonate ( Barde *et al.*, 2024). The incident affected over 10 km of creeks lying near the exploration mine. The river contained high concentrations of iron and manganese deriving from the presence of iron ore on the plateau ( Isinkaye *et al.*, 2023).

## 7. Heavy metal pollution mitigation strategies

Mitigation of heavy metal bioaccumulation, contamination and pollution is very much imperative in terms of the environment, as widespread pollution increases human exposure and will definitely cause debilitating effects on the human body. Several strategies exist for the decontamination and removal of heavy metals from both the environment and the human body ( Wang *et al.*, 2022a; Kowalczyk *et al.*, 2021).

Since the discovery of the problem of environmentally accumulated heavy metals, various technologies have been developed to reduce or eliminate the concentration of heavy metals that have polluted the soil and water. The three main technologies are in situ stabilization and phytoremediation in the environment and chelation therapy to reduce metal-induced oxidative stress, signal transduction, and organ-specific physiology within human beings ( Kumar *et al.*, 2023). *’In situ’* refers to on-site or on-area-site and can be accomplished without removing soil from contaminated sites. Chelation is a demure and direct strategy for scavenging the excess metal ions in vital organs of the body, which shows excellent results in flu treatment. In the environmental field, research focusing on bioaccumulation of metals by plants led to the development of technologies to extract heavy metals from the environment, so-called phytoremediation. Phytoremediation is a green technology and is a site-specific technology, which can ultimately reduce the risk of human exposure to toxic substances and environmental remediation costs. Phytoremediation technology includes phytoextraction, phytodegradation, phytostabilization, rhizofiltration, phytovolatilization, phytostimulation, rhizodegradation, rhizofiltration, and hyperaccumulation ( Lee and Lee, 2024; Ali, 2023; Chen and Costa, 2021).

The term phytoremediation is used to describe a set of processes involving the ability of certain plants to remove, degrade, or immobilize a variety of contaminants present in the environment so they are no longer harmful ( Yaashikaa *et al.*, 2022). In the case of heavy metals, the term was coined by the US EPA in the early 90s to define the “use of green plants to remove pollutants from the environment or render them harmless”. Phytoremediation is being envisaged as a sustainable approach for in situ remediation of contaminated soils, sediments, and groundwater, due to economic and environmental considerations. This technology can be used to reduce the level of metals in a toxic range for human health and greatly reduce the uptake of these contaminants by plants. In fact, it seems to be effective in plant-only systems, where metals do not accumulate in the food chain, and whenever proper disposal procedures for the harvested material are followed. Since the steel industry is a primary source of contamination by metals and metalloids like chromium, lead, and zinc, good results in terms of removal, stabilization, or reduction can be of interest. In particular, phytoremediation technology appears to be particularly suitable for agricultural substrates or in regions with a view to re-cultivation. Taken together it is very much imperative that safe phytoremediation technology is used to avoid potential harm.

Chelation therapy is a therapeutic intervention in which chelating agents are administered to individuals for the removal of toxic heavy metals from the body ( Bjørklund *et al.*, 2020). Chelating agents are essentially metal binders and remove them from their sites of deposition in the tissues, in this way, the heavy metal toxins are eliminated in the urine ( Gerhardsson, 2022). There are two routes of administration, namely by the oral and parenteral routes, of which the parenteral route is the most effective. Chelation therapy is of benefit in cases of lead and mercury poisoning ( Angle, 2023). Chelation could prevent various toxicological manifestations of heavy metals, thus markedly improving various pathophysiological functions. Chelation therapy has been shown to have successful outcomes in cases of lead overload in patients such as children and pregnant women ( Singh *et al.*, 2021). However, chelation therapy is ineffective in treating advanced malignancies ( Kontoghiorghes, 2022). Despite such knowledge, chelation therapy has also been reported to be effective in the treatment of other heavy metals in the environmental setting ( Glicklich *et al.*, 2020).

Another most common use of chelation therapy is in the treatment of cardiac and cerebral vascular ailments ( Ravalli *et al.*, 2022). This therapy is aimed at removing the vascular oxidative injury caused by superoxide and hydroxyl anions by binding these mobilized metal ions (Fe2+ and Ca2+) ( Kontoghiorghes *et al.*, 2020). Lead poisoning is the most common cause of neuropathy, and it usually requires an individual to undergo chelation therapy to excrete lead out ( Bhasin *et al.*, 2023).

## 8. Conclusion

In conclusion, heavy metals are major sources of contamination and have adverse effects on human health when their levels exceed the regulatory standards ( Mishra *et al.*, 2019). According to the available literature data, if direct human exposure to heavy metal residues in food, water, and/or air occurs, it can lead to severe health problems, e.g., anemia, cancer, or kidney disease ( Briffa et *al.*, 2020; Balali-Mood *et al.*, 2021). The data in the literature reviewed for this article have shown that there are multiple sources and pathways of exposure to toxic heavy metals in our environment and identifying the exact causes and adverse effects of contamination on human health is very difficult ( Briffa *et al.*, 2020; Bhardwaj *et al.*, 2021; Mitra *et al.*, 2022). Moreover, the removal of metals from the body appears to be limited and slow and may depend on age, sex, and individual genetic variation. In this regard, additional studies are required to find an effective, quick, and safe method for their removal.

Indeed, the need to address heavy metal contamination is very much imperative for a healthy environment and public health. The elements that are classified as heavy metals are difficult to degrade or decompose with time. Additionally, these toxic heavy metals can also flow through the food web and bioaccumulate in various tissues of humans and animals over time due to dietary habits ( Nkwunonwo *et al.*, 2020: Sonone *et al.*, 2020). Overall, several challenges, including health risk assessments to determine potential health risks associated with heavy metals, bioavailability assessment to predict the potential for heavy metal bioaccumulation and toxicity in humans, and reducing heavy metal intake, overclocking through legislations to ensure enforcement of existing and future regulatory methods, and encouraging food production in uncontaminated areas, must be addressed by scientists, regulatory authorities, and stakeholders in order to protect human health. In view of these analyses, further research studies should focus on tracing the sources of heavy metals in the environment to find effective ways to overcome this contamination. Moreover, future practices and policies should focus on the development and implementation of economic strategies to reduce or control contamination with toxic heavy metals.

### Ethics and consent

Ethics and consent were not required.

## Data Availability

No data are associated with this article.
