# Supplementary material for: Heavy Metal Contamination: Sources, Health Impacts, and Sustainable Mitigation Strategies with Insights from Nigerian Case Studies
Source: F1000Res. 2025 Jul 11;14:134. Originally published 2025 Jan 27. [Version 4] doi: 10.12688/f1000research.160148.4 (PMC12355173; doi:10.12688/f1000research.160148.4)
Supplement: Peer review history

# Reviewer report: Reviewer response for version 4

**DOI:** 10.5256/f1000research.184191.r398249

**Published:** 2025-08-14

**Authors:** Rupesh Kumar

**Affiliations:** 1 O P Jindal Global University, Sonipat, Haryana, India

Accepting this paper in current form.

Is the review written in accessible language?

Yes

Are all factual statements correct and adequately supported by citations?

Yes

Are the conclusions drawn appropriate in the context of the current research literature?

Yes

Is the topic of the review discussed comprehensively in the context of the current literature?

Yes

Reviewer Expertise:

NA

I confirm that I have read this submission and believe that I have an appropriate level of expertise to confirm that it is of an acceptable scientific standard.

# Reviewer report: Reviewer response for version 3

**DOI:** 10.5256/f1000research.181015.r383061

**Published:** 2025-06-16

**Authors:** Muyiwa Michael Orosun

**Affiliations:** 1 University of Ilorin, Ilorin, Kwara, Nigeria

The introduction provides a comprehensive overview of heavy metal contamination, its sources, and health impacts, effectively setting the stage for the review. However, the abstract could be more concise and structured to clearly highlight the study's objectives, key findings, and implications. The authors should explicitly state the novelty of their work, such as integrating Nigerian case studies with global comparisons, to emphasize its contribution to the field. Additionally, the introduction would benefit from a clearer articulation of research gaps, such as the lack of longitudinal studies on heavy metal exposure in Nigeria or the need for advanced predictive modeling. The authors should also ensure that all references cited in the text are listed in the reference section and vice versa, as discrepancies undermine credibility.

 The methodology section is implicit rather than explicit, which is common in narrative reviews but could be strengthened. The authors should clarify their literature search strategy, including databases used, keywords, and inclusion/exclusion criteria. A systematic approach would enhance reproducibility. For future work, the authors could consider employing Monte Carlo techniques to model the probabilistic health risks associated with heavy metal exposure, particularly in Nigerian case studies. Check the works of Orosun et al and Isinkaye et al for a good grasp. This would add a quantitative dimension to their qualitative analysis. Additionally, the reference formatting should be standardized (e.g., ensuring journal names are italicized and consistent with the target journal’s style).

 The results and discussion are detailed, particularly in describing health impacts and Nigerian case studies. However, the global comparison section (e.g., Pakistan, India) feels underdeveloped. The authors should expand this to highlight parallels and unique challenges in Nigeria. The discussion on mitigation strategies (e.g., phytoremediation, chelation) is thorough but could be enriched with cost-benefit analyses or success rates from other regions. The authors should also ensure that all in-text citations match the reference list and that each reference is formatted correctly (e.g., consistent use of "et al." and proper abbreviation of journal names). A table summarizing case studies and their outcomes would improve readability.

 The conclusion effectively summarizes key points but could be more forward-looking. The authors should explicitly recommend policy actions (e.g., stricter mining regulations) and research priorities (e.g., epigenetic studies or AI-driven monitoring). The reference section is extensive but requires meticulous verification. 

 Encouraging the use of Monte Carlo simulations for risk assessment in future studies would strengthen the manuscript’s methodological rigor. Minor grammatical errors (e.g., "sip into soil" corrected to "seep") should also be addressed in a final proofread.  

 Overall, the manuscript is well-researched but would benefit from a tighter structure, clearer novelty, and methodological transparency to elevate its impact.

Is the review written in accessible language?

Yes

Are all factual statements correct and adequately supported by citations?

Yes

Are the conclusions drawn appropriate in the context of the current research literature?

Yes

Is the topic of the review discussed comprehensively in the context of the current literature?

Yes

Reviewer Expertise:

Environmental Health

I confirm that I have read this submission and believe that I have an appropriate level of expertise to confirm that it is of an acceptable scientific standard, however I have significant reservations, as outlined above.

# Reviewer report: Reviewer response for version 2

**DOI:** 10.5256/f1000research.178309.r372442

**Published:** 2025-04-12

**Authors:** Qudrat Ullah

**Affiliations:** 1 Agriculture and Food Science, Walailak University (Ringgold ID: 65133), Thai Buri, Nakhon Si Thammarat, Thailand

Summary of the Article

 The review article by Laoye et al. explores the critical issue of heavy metal contamination, focusing on its sources, health impacts, and sustainable mitigation strategies, with a particular emphasis on case studies from Nigeria. The authors discuss heavy metals such as lead, mercury, cadmium, and arsenic, detailing their natural and anthropogenic origins, including geological processes and human activities like industrial emissions, mining, and agriculture. The article highlights how these metals enter environmental systems (soil, water, and food chains), leading to bioaccumulation and biomagnification, which pose significant health risks, including cardiovascular diseases, neurological disorders, and cancers. Nigerian case studies, such as lead poisoning in Zamfara State and contamination in the Great Kwa River, provide region-specific insights into the socio-economic and environmental impacts of heavy metal pollution. The review evaluates mitigation strategies like chelation therapy and phytoremediation, advocating for integrative approaches involving policy, community engagement, and technological innovation. The authors conclude that understanding heavy metal toxicity and implementing sustainable solutions are vital for environmental management and public health.

 Detailed Assessment and Recommendations

1. **Comprehensiveness in the Context of Current Literature (Partly)**
  The article provides a broad overview of heavy metal contamination, covering sources, health impacts, exposure routes, toxicity mechanisms, and mitigation strategies. It effectively integrates Nigerian case studies, which add contextual relevance and highlight challenges in developing nations. The inclusion of recent references (up to 2024) demonstrates engagement with current literature, and the discussion of specific heavy metals (lead, mercury, cadmium, arsenic) is detailed and well-supported.
  However, the review could be more comprehensive in several areas:
  - **Global Context**: While the Nigerian case studies are valuable, the article could strengthen its global perspective by comparing these cases with similar incidents in other regions (e.g., Minamata Bay for mercury or arsenic contamination in Bangladesh). This would enhance the universality of the findings and situate the Nigerian experience within a broader framework.
  - **Emerging Research Areas**: The review briefly mentions oxidative stress and DNA damage as toxicity mechanisms but does not explore newer research areas, such as epigenetic effects or the role of the microbiome in heavy metal toxicity. Including these could make the review more forward-looking.
  - **Mitigation Technologies**: The discussion of phytoremediation and chelation therapy is adequate but lacks depth on emerging technologies like nanotechnology or bioremediation using genetically modified organisms. Expanding this section would align the article with cutting-edge developments.
  **Recommendations**:
  - Add a subsection comparing Nigerian case studies with global examples to provide a more comprehensive context.
  - Include recent studies on emerging toxicity mechanisms (e.g., epigenetic changes) and cite relevant literature (e.g., studies from 2023–2024 on heavy metal epigenetics).
  - Expand the mitigation section to cover novel technologies, such as nanomaterial-based remediation, with appropriate citations.
2. **Accuracy and Support of Factual Statements (Yes)**
  The factual statements in the article appear correct and are well-supported by citations from reputable sources. The authors draw on a wide range of studies (e.g., WHO, 2023; Genchi et al., 2020; Mitra et al., 2022) to substantiate claims about heavy metal sources, health impacts, and mitigation strategies. The Nigerian case studies are grounded in specific incidents (e.g., Zamfara lead poisoning), and the cited references align with the claims made.
**Observation**: While the citations are robust, some sections (e.g., mechanisms of toxicity) rely heavily on older references (e.g., Shi et al., 2004). Incorporating more recent studies would strengthen the article’s currency.
**Recommendation**:
  - Review sections like “Mechanisms of Heavy Metal Toxicity” and update with citations from 2023–2024 to reflect the latest understanding of oxidative stress, DNA damage, or other mechanisms.
3. **Accessibility of Language (Yes)**
  The review is written in clear, accessible language suitable for a broad scientific audience, including researchers, policymakers, and environmental scientists. Technical terms like “bioaccumulation,” “phytoremediation,” and “oxidative stress” are used appropriately and explained implicitly through context, making the article approachable without oversimplification. The structure, with clear headings (e.g., “Sources of Heavy Metal Contamination,” “Health Impacts”), aids readability.
**Observation**: Minor typographical errors and awkward phrasing (e.g., “sip into soil” instead of “seep into soil” on Page 1) slightly detract from the polished presentation.
**Recommendation**:
  - Conduct a thorough proofreading to correct minor errors and improve sentence flow (e.g., revise “sip into soil” to “seep into soil” and “very much important significant” to “highly significant” on Page 1).
4. **Appropriateness of Conclusions (Partly)**
  The conclusions emphasize the need for integrative approaches to combat heavy metal contamination, highlighting policy, community engagement, and technological innovation. These are appropriate given the evidence presented and align with the literature’s call for multidisciplinary solutions. The focus on Nigerian case studies supports the conclusion’s emphasis on region-specific vulnerabilities.
  However, the conclusions could be strengthened in the following ways:
  - **Specificity**: The conclusion is somewhat general, stating the importance of “sustainable environmental management practices” without detailing actionable steps or prioritizing strategies based on the Nigerian context. For example, it could specify whether phytoremediation or policy enforcement is more feasible in resource-constrained settings like Nigeria.
  - **Comparison with Other Studies**: The conclusion does not explicitly compare the findings with other reviews, missing an opportunity to highlight what this study uniquely contributes (e.g., the Nigerian case studies).
  - **Limitations**: While Version 2 mentions limitations, the conclusion does not fully address how these (e.g., lack of discussion on certain technologies) impact the proposed strategies.
  **Recommendations**:
  - Revise the conclusion to include specific, prioritized recommendations for Nigeria, such as strengthening regulatory frameworks for mining or scaling up phytoremediation pilot projects.
  - Compare the review’s findings with other studies to clarify its unique contributions (e.g., how the Nigerian focus adds to global heavy metal research).
  - Explicitly address limitations in the conclusion, such as the scope of mitigation strategies discussed, and suggest how future research could fill these gaps.

Additional Comments

- **Nigerian Case Studies**: The inclusion of Zamfara and Great Kwa River case studies is a strength, but more detail on their outcomes (e.g., current status, long-term health impacts, or remediation efforts) would enhance their impact. For instance, what lessons from Zamfara have informed national policy?
- **Structure**: The article is well-organized, but a dedicated section on research gaps and future directions (beyond the brief mention in the conclusion) would add value. This could outline unanswered questions, such as the long-term efficacy of phytoremediation in tropical climates.
- **Response to Previous Review**: The authors’ response to Reviewer 1 (Rupesh Kumar) shows they addressed concerns like revising the conclusion and noting limitations. However, their dismissal of alternative technologies (e.g., AI predictive models) as outside the scope seems limiting. While not central, a brief mention of data-driven approaches for monitoring contamination could broaden the mitigation discussion.

Points to Address for Scientific Soundness

 To ensure the article is scientifically sound, the authors must:

1. Enhance comprehensiveness by comparing Nigerian case studies with global examples and including emerging research areas (e.g., epigenetics, novel remediation technologies).
2. Update citations in the toxicity mechanisms section with recent studies to maintain currency.
3. Revise the conclusion to provide specific, actionable recommendations and explicitly address limitations, ensuring alignment with the evidence presented.
4. Proofread the manuscript to eliminate minor errors and improve clarity.

Approval Status

**Approved with Reservations**

 The article is a valuable contribution to the literature on heavy metal contamination, particularly for its focus on Nigerian case studies and integration of health and mitigation perspectives. However, addressing the above recommendations will improve its comprehensiveness, specificity, and alignment with current research, making it more robust and impactful.

Is the review written in accessible language?

Yes

Are all factual statements correct and adequately supported by citations?

Yes

Are the conclusions drawn appropriate in the context of the current research literature?

Partly

Is the topic of the review discussed comprehensively in the context of the current literature?

Partly

Reviewer Expertise:

As Qudrat, I specialize in environmental toxicology and public health, with a focus on the impacts of environmental contaminants, such as heavy metals, on human health and ecosystems. My expertise includes assessing sources and pathways of contamination, evaluating toxicological mechanisms (e.g., oxidative stress, bioaccumulation), and reviewing mitigation strategies like phytoremediation and policy interventions. I am well-equipped to evaluate the scientific validity of the article’s discussion on heavy metal sources, health effects, and mitigation approaches, as well as its use of Nigerian case studies to highlight regional vulnerabilities. However, I may have limited capacity to assess highly specialized aspects outside my scope, such as advanced biochemical modeling or cutting-edge nanotechnology applications, though these are not central to the article’s current content.

I confirm that I have read this submission and believe that I have an appropriate level of expertise to confirm that it is of an acceptable scientific standard, however I have significant reservations, as outlined above.

## References

[1] : Water Quality Degradation Due to Heavy Metal Contamination: Health Impacts and Eco-Friendly Approaches for Heavy Metal Remediation. *Toxics* .2023;11(10) : 10.3390/toxics11100828 10.3390/toxics11100828 37888679 PMC10611083

[2] : Assessment of heavy metal contamination levels and health risks in environmental media in the northeast region. *Sustainable Cities and Society* .2022;80: 10.1016/j.scs.2022.103796 10.1016/j.scs.2022.103796

# Response

**Published:** 2025-04-22

**Authors:** Babafemi Laoye

**Affiliations:** Biological Sciences, Afe Babalola University, Ado Ekiti, Ekiti, Nigeria

**Reviewer’s comments**

**Recommendations**:

- Add a subsection comparing Nigerian case studies with global examples to provide a more comprehensive context.
- Include recent studies on emerging toxicity mechanisms (e.g., epigenetic changes) and cite relevant literature (e.g., studies from 2023–2024 on heavy metal epigenetics).
- Expand the mitigation section to cover novel technologies, such as nanomaterial-based remediation, with appropriate citations.

**Authors’ response**

 This has been harmonized and well attend to in the manuscript.

**Reviewers’ comments**

**Recommendation**:

 Review sections like “Mechanisms of Heavy Metal Toxicity” and update with citations from 2023–2024 to reflect the latest understanding of oxidative stress, DNA damage, or other mechanisms

**Authors’ response**

 This has been harmonized, citations from 2023-2024 has been added

**Reviewers’ comments**

**Recommendation**:

- Conduct a thorough proofreading to correct minor errors and improve sentence flow (e.g., revise “sip into soil” to “seep into soil” and “very much important significant” to “highly significant” on Page 1).

**Authors’ response**

 The correction has been made and well harmonized

**Reviewers’ comments**

**Recommendations**:

- Revise the conclusion to include specific, prioritized recommendations for Nigeria, such as strengthening regulatory frameworks for mining or scaling up phytoremediation pilot projects.
- Compare the review’s findings with other studies to clarify its unique contributions (e.g., how the Nigerian focus adds to global heavy metal research).
- Explicitly address limitations in the conclusion, such as the scope of mitigation strategies discussed, and suggest how future research could fill these gaps.

**Authors’ response**

 This has been well harmonized

**Reviewers’ comments**

 Additional Comments

- Nigerian Case Studies: The inclusion of Zamfara and Great Kwa River case studies is a strength, but more detail on their outcomes (e.g., current status, long-term health impacts, or remediation efforts) would enhance their impact. For instance, what lessons from Zamfara have informed national policy?
- Structure: The article is well-organized, but a dedicated section on research gaps and future directions (beyond the brief mention in the conclusion) would add value. This could outline unanswered questions, such as the long-term efficacy of phytoremediation in tropical climates.
- Response to Previous Review: The authors’ response to Reviewer 1 (Rupesh Kumar) shows they addressed concerns like revising the conclusion and noting limitations. However, their dismissal of alternative technologies (e.g., AI predictive models) as outside the scope seems limiting. While not central, a brief mention of data-driven approaches for monitoring contamination could broaden the mitigation discussion.

**Authors’ response**

 This has been addressed and harmonized in the revised manuscript. A brief mention of Ai data driven approaches has been duly mention and harmonized in the conclusion of the revised manuscript

**Reviewers’ comments**

- Points to Address for Scientific Soundness
  To ensure the article is scientifically sound, the authors must:
- Enhance comprehensiveness by comparing Nigerian case studies with global examples and including emerging research areas (e.g., epigenetics, novel remediation technologies).
- Update citations in the toxicity mechanisms section with recent studies to maintain currency.
- Revise the conclusion to provide specific, actionable recommendations and explicitly address limitations, ensuring alignment with the evidence presented.
- Proofread the manuscript to eliminate minor errors and improve clarity.

**Authors’ response**

 All the corrections emphasized has been made and incorporated in the revised manuscript.

# Reviewer report: Reviewer response for version 1

**DOI:** 10.5256/f1000research.175987.r363027

**Published:** 2025-02-08

**Authors:** Rupesh Kumar

**Affiliations:** 1 O P Jindal Global University, Sonipat, Haryana, India

Few important observations needs to be addressed for improving the quality of work:

 1) What is the novelty of study? Is there any existing work supporting or motivating to do this study? Please explain in Introduction part.

 2) Author should also mention some other existing technologies may be considered or used instead of AI predictive.

 3) I unable to see the research gaps and objectives. Please include latest references which will give you an idea of work in different sectors. Few important papers needs to be added:

 i. Kumar R et al. (2014 [Ref-1]) 

 ii. Rajani R et al. (2022 [Ref-2])

 iii. Kumar R (2017 [Ref-3]) 

 4) Methodology: Please check for proper citation of figures and tables in the text. In addition, there is no source given for figure and tables. If it is self then author should cite as “Source: Author Composition” or similar.

 5) Implications should be added in this (Political, Practical and Social if any).

 6) Conclusion part is written in very short. Compare your study with other authors and write in conclusion and extend a bit more.

 7) Limitations and future work should be included as a separate heading or may be a part of conclusion also in the last para.

 8) Please make a proper structure of your manuscript like aligning with Introduction, LR, Gaps, Objectives, Methods and Materials, Analysis, Results and Discussion, Implications, Conclusion, Limitations and Future Scope and References.

 9) Authors may proof read before submitting the revision.

Is the review written in accessible language?

Yes

Are all factual statements correct and adequately supported by citations?

Yes

Are the conclusions drawn appropriate in the context of the current research literature?

Yes

Is the topic of the review discussed comprehensively in the context of the current literature?

Yes

Reviewer Expertise:

Same area

I confirm that I have read this submission and believe that I have an appropriate level of expertise to confirm that it is of an acceptable scientific standard, however I have significant reservations, as outlined above.

## References

[1] : Barriers to E-Application in Agrifood Supply Chain.2014; 10.4018/978-1-4666-5202-6.ch022 227-240 10.4018/978-1-4666-5202-6.ch022

[2] : Demand management strategies role in sustainability of service industry and impacts performance of company: Using SEM approach. *Journal of Cleaner Production* .2022;369: 10.1016/j.jclepro.2022.133311 10.1016/j.jclepro.2022.133311

[3] : Multi-criteria decision and multivariate statistical approaches improve olive supply chains: a review. *International Journal of Value Chain Management* .2017;8(3) : 10.1504/IJVCM.2017.086838 10.1504/IJVCM.2017.086838

# Response

**Published:** 2025-02-14

**Authors:** Babafemi Laoye

**Affiliations:** Biological Sciences, Afe Babalola University, Ado Ekiti, Ekiti, Nigeria

The authors are grateful for the feedback received from the reviewer. Where necessary, appropriate amendments have been made in the manuscript. Below are responses to the review queries:

**Reviewer’s query:**  What is the novelty of the study? Is there any existing work supporting or motivating to do this study? Please explain in the introduction part.

**Authors’ response:** Although there are a plethora of research studies that have reported the debilitating effects of heavy metals on the health due to contamination from various sources, the focus of this review was that, apart from discussing the health impacts of heavy metal contamination, case studies in the Nigerian environmental context were discussed, which was a departure from existing literature. This is clearly embedded in the introduction.

**Reviewer’s query**: Author should also mention some other existing technologies that may be considered or used instead of AI predictive

**Authors’ response:** The focus of the review did not include discussion on existing technologies. This could be a limitation of the review

**Reviewer’s query**: I am unable to see the research gaps and objectives. Please include the latest references, which will give you an idea of work in different sectors. A few important papers need to be added.

 i. Kumar R et al. (2014 [Ref-1]) 

 ii. Rajani R et al. (2022 [Ref-2])

 iii. Kumar R (2017 [Ref-3]) 

**Authors’ response**: Thank you for your kind observation; the research gap has been included, although the authors agree that there are a plethora of recent references included in this manuscript and the case studies in this manuscript reflect different sectors. In as much as the authors were willing to cite the aforementioned papers, perusing them revealed their focus did not align with the scope of this manuscript.

**Reviewer’s query:** Methodology: Please check for proper citation of figures and tables in the text. In addition, there is no source given for figures and tables. If it is self, then the author should cite as “Source: Author Composition” or similar.

**Authors’ response**: All citations have been checked. However, the manuscript contained neither tables nor figures.

**Reviewer’s query:** Implications should be added in this (Political, Practical and Social if any).

 Authors’ response; Attended to and well noted.

**Reviewer’s query:** Conclusion part is written in very short. Compare your study with other authors and write in conclusion and extend a bit more.

 Authors’ response: The conclusion has been re-written.

**Reviewer’s query:** Limitations and future work should be included as a separate heading or may be a part of the conclusion also in the last paragraph.

 Authors’ response: That has been attended to.

**Reviewer’s query**: Please make a proper structure of your manuscript like aligning with Introduction, LR, Gaps, Objectives, Methods and Materials, Analysis, Results and Discussion, Implications, Conclusion, Limitations and Future Scope and References.

**Authors’ response:** The suggested structure would have been appropriate if it were a research article. There is no way that structure can be followed for a review manuscript as in this case. A review paper (except it is a systematic review) cannot follow the conventional format.

**Reviewer’s query**: Authors may proof read before submitting the revision.

**Authors’ response**: That has been attended to and well noted.
